# A Treatise on the Principles and Practice of Medicine, Designed for the Use of Practitioners and Students of Medicine

**Published:** 1866-08

**Authors:** 


### BIBLIOGRAPHICAL.

*A Treatise on the Principles and Practice of Medicine, designed for the use of Practitioners and Students of Medicine.* By AUSTIN FLINT, M. D.

The above is the title of a work before us, received through the courtesy of the publisher, Henry C. Lea, Philadelphia, from the pen of the eminent Professor of the Principles and Practice of Medicine in the Bellevue Hospital Medical College, New York. The volume contains 867 pages, and is divided into two parts, the first of which contains about one-eighth of the matter and is devoted to the Principles of Medicine, or General Pathology, and the remainder and bulk of the work is devoted to Special Pathology, or the Practice of Medicine.

These divisions are preceded by an introductory chapter which contains a brief but lucid reference to the following points: Scope of the term Medicine; use of the term in contradistinction to surgery and obstetrics; subdivisions of the different departments of Medicine or Specialities; the general object of the work; meaning of the phrase Principles and Practice of Medicine; definition of Pathology; division into General and Special Pathology; nomenclature of diseases; subdivisions of General Pathology, viz.: morbid anatomy, and morbid changes of the fluids of the body; etiology; symptomatology; diagnosis; prognosis; prophylaxis and therapeutics; relations of these subdivisions to Special as well as General Pathology; definition of disease; definition of health; relationship of Pathology to Physiology; progress of pathological knowledge.

Part 1st, or that portion of the work devoted to the Principles of Medicine, or General Pathology, contains three chapters upon the Anatomical Changes in the solid parts of the body, three upon the Morbid Conditions of the Blood, and one each, upon the

Causes of Disease or Etiology, Symptomatology, and General Therapeutics. Part 2d, or the Treatise upon the Practice of Medicine, or Special Pathology, is divided into six sections, the first of which is divided into eleven chapters embracing the Special Pathology and treatment of pleuritis, pneumonitis, bronchitis, emphysema, asthma, hemorrhagic affections, affections of the larynx and trachea and pulmonary tuberculosis. The 2d treats exclusively of diseases affecting the circulatory system, and inflammatory affections of the heart; valvular lesions with enlargement of the heart, and enlargement of the heart without valvular lesions. Section 3d is devoted to diseases affecting the digestive system, with separate chapters on inflammatory affections of the alimentary canal; structural affections of the stomach; structural affections of the intestinal canal, involving obstructions; functinal affections of the stomach and intestines; cholera; intestinal worms; inflammation of the peritoneum, and peritoneal dropsy; diseases affecting the collatitious or solid viscera of the abdomen.

Section 4th is made up of ten chapters devoted to diseases affecting the nervous system and respectively to inflammatory affections of the meninges of the brain and spinal cord; inflammatory affections and structural lesions of brain and spinal cord; inflammatory paralysis, and the neuroses, viz: neuralgia, chorea, epilepsy, hysteria, catalepsy, ecstacy, somnambulism, tetanus, rabies, delirium tremens, alcoholism and nervous asthenia.

Section 5th is appropriated to diseases affecting the genito-urinary system, with chapters on inflammatory affections of the kidneys, structural affections of the kidneys, and diseases of the supra-renal capsules, Addison's disease; involuntary seminal emissions, spermatorrhœa and impotence being included under the latter head.

Section 6th contains twelve chapters devoted to fevers and other general diseases, with special observations upon continued fevers; periodical fevers, diphtheria; acute articular rheumatism; gout and scorbutus, or scurvy.

The object of the work, as set forth in the preface, is to present such a digest of the Principles and Practice of Medicine as will be alike serviceable to the pupil in the prosecution of his studies of disease, and to the physician engaged in the practical duties of his profession. Very little space is occupied with past opinions or doctrines which have become obsolete. Discussions relating to mooted pathological questions are barely entered into, and subjects belonging to other departments of instruction are for the most part omitted; hence for information on matters relating to surgery, obstetrics, the diseases of women and children, cutaneous diseases, and the details of the Materia Medica, the reader is referred to other works, and the object of the volume seems to have been well sustained in keeping prominently in mind the practical applications of medical knowledge to Diagnosis, Prophylaxis and Therapeutical indications.

Upon a careful examination of this work, we have no hesitation in recommending it as one more nearly representing the existing state of the science and art of medicine, and more nearly reflecting the views of those who exemplify in their practice the present stage of a progressive pathology, than any other American book upon Practical Medicine. As illustrative of the views to which we allude and desire specially to commend and of the style of the work, we insert the following extract upon the subject of bloodletting.

"A great change has taken place within the last few years, with respect to bloodletting in the treatment of acute inflammations. This measure was formerly thought to be highly important, and was rarely omitted. It is now considered by many as seldom, if ever, called for. The infrequent use of the lancet now, contrasted with its frequent use twenty-five years ago, constitutes one of the most striking of the changes in the practice of medicine which have occurred during this period. It can hardly be doubted that this measure was formerly adopted too indiscriminately, and often employed too largely; but, with the natural tendency to pass from one extreme to another, it may be that the utility of bloodletting in certain cases, at the present time, is not sufficiently appreciated.

Experience and pathological reasoning combine to show that bloodletting does not exert a direct controlling effect upon an inflammatory disease. It may exert a powerful immediate effect as a palliative measure, and whatever curative power it may possess is exerted indirectly. Its therapeutic action consists in lessening the frequency and force of the heart's action; in other words, in diminishing the intensity of symptomatic fever. In the early period of an acute inflammation accompanied by high febrile movement, as indicated by a pulse accelerated and of abnormal strength, the abstraction of blood affords relief, and may contribute to a favorable progress of the disease. It should enter into the treatment of a certain proportion of cases, provided other and more conservative means for the same ends are not available.

The evils of bloodletting arise from its spoliative effect upon the blood. It diminishes the red corpuscles, and these, during the progress of an acute disease, are not readily reproduced. It induces, thus, the anæmic condition, and in this way impairs the vital powers. It will be likely to do harm, therefore, whenever it is important to economize the powers of life, and it may contribute to a fatal result in diseases, or cases of disease, which involve danger of death by asthenia.

The useful effects of bloodletting may frequently, if not generally, be obtained by other means which require less circumspection in their employment, because, if injudiciously resorted to, they are in a less degree hurtful. The mass of blood may be temporarily lessened by saline purgatives and diaphoretic remedies, conjoined with a restricted ingestion of food and liquids. Depletion is obtained in this way without spoliation or impoverishment of the blood. The frequency and force of the heart's action may be affected by nauseant sedatives such as tartar-emetic, ipecacuanha, etc., and by direct sedatives, viz: digitalis, aconite, and the veratrum viride. By saline depletories and sedatives, the symptomatic fever may be modified without the expenditure of blood, and thus the evils of bloodletting avoided. The advantage of bloodletting consists mainly in the promptness of its operation. Several hours are required to secure results from the means employed in lieu of bloodletting, while the effects of the latter are produced in a few moments.

In accordance with these views, bloodletting is never indicated by the fact simply that acute inflammation exists; it is a measure directed, not to the disease *per se*, but to circumstances associated with the disease. The state of the circulation, and other circumstances, furnish the indications for the employment of this measure. It is admissible if, with the development of inflammation, there exist high symptomatic fever, the pulse denoting augmented power of the heart's action; the patient robust and in good health when attacked, and the disease not involving danger of death by asthenia. The measure is admissible under the conditions just stated, whenever the promptness with which its effects are obtained renders it desirable to adopt it in preference to other measures producing the same effects with some delay. *Per contra*, bloodletting is not admissible when the development of inflammation is not accompanied by high symptomatic fever, and the pulse does not indicate augmented power of the heart's action ; nor when the patient was not in good health when attacked, nor when the constitution is feeble, nor when the disease involves danger of death by asthenia. These rules of practice, while they accord to bloodletting therapeutic value, undoubtedly restrict its use within narrow limits.

Applying these rules to the disease under consideration, a patient in the first stage of acute pleuritis, robust, perhaps plethoric, suffering from severe pain and a sense of oppression, with a strong non-compressible pulse, will derive immediate relief from the abstraction of from ten to sixteen ounces of blood. The loss of this quantity of blood under such circumstances, in a disease like this which does not tend to destroy life by asthenia, will give rise to no evil results, but will be likely to affect favorably the progress of the disease. On the other hand, a patient feeble or perhaps anæmic, with a pulse denoting excited action, not increased power, should not be bled, notwithstanding the local symptoms would undoubtedly be thereby relieved. By impairing the vital powers, the loss of blood will do harm, and is not admissible, under these circumstances, merely as a palliative remedy. And, in the first case, if the local symptoms do not urgently call for immediate relief, other measures may be substituted for the bloodletting.

Before leaving the consideration of bloodletting, several incidental points may be briefly noticed.

This measure is perhaps more applicable to the treatment of inflammation affecting the pulmonary organs than to the treatment of other inflammatory affections, in consequence of the relations of the former to the circulation. The free passage of the blood through the pulmonary circuit seems to be promoted, and the functional labor which the lungs perform is diminished by the abstraction of blood. At all events, relief of the sense of oppression and dyspnœa attendant on the early stage of acute inflammation of any of the pulmonary structures is more quickly and effectually procured by bloodletting than by other measures. Were it not for its ulterior effects, it would be invaluable as a palliative measure in pleurisy and other inflammatory affections within the chest.

The evils of indiscriminate and excessive bloodletting are manifested by a larger rate of mortality in those diseases which tend to destroy life by asthenia, and it can hardly be doubted that the death-rate has been diminished by a much more sparing use of the lancet within late years. But the results of injudicious bloodletting are manifested in cases which end in recovery, as well as those which end fatally. These results consist in a protracted convalescence and subsequent feebleness. The cases of different inflammations treated formerly by bloodletting and other measures entering into the so-called antiphlogistic method, and the cases now treated otherwise, present a striking contrast as regards the condition of patients during convalescence and after recovery.

The opinion is held by some that diseases, and the human constitution, have undergone a notable change during the last quarter of a century, and that bloodletting and other antiphlogistic measures are less appropriate now than formerly, on this account. This opinion seems to me not well founded. After a professional experience extending beyond the period just named, I do not hesitate to express a conviction that acute inflammations at the present day are essentially the same as they were twenty-five years ago, and that antiphlogistic measures were no more appropriate then than now.

Were it true that such changes have occurred, the fact would strike at the root of medical experience. If changes requiring a revolution in therapeutics are liable to occur with each successive generation, it is evident there can be no such thing as permanent principles of practice in medicine; the fruits of experience in our day, which so many are striving to develope, will be of no utility to those who are to come after us.

In addition to general bloodletting, or the employment of venesection, much importance was formerly attached to the abstraction of blood by cups or leeches applied in the neighborhood of the inflamed part. Local bloodletting, in some cases, is more convenient than general; but, so far as the abstraction of blood is concerned, it is difficult to conceive that it is a matter of much importance from what part of the body or vascular system it is taken. Whether it be abstracted by means of cups, leeches, or the lancet, the benefit or injury will depend on the quantity withdrawn in a given period. Whatever advantage may accrue from the removal of a certain amount of blood by cups or leeches, over the abstraction of the same amount by venesection, must be derived from the operation of the former as revulsive measures."

Upon the whole we would most earnestly recommend Dr. Flint's work to the profession as possessing claims to merit of the highest order.

